# Anxiety, depression, and perceived wellbeing in antenatal women at risk of preterm birth: a retrospective cohort study

**DOI:** 10.3389/fgwh.2024.1511352

**Published:** 2024-12-06

**Authors:** Semra Worrall, Paul Christiansen, Naomi Carlisle, Victoria Fallon, Asma Khalil, Andrew H. Shennan, Rachel M. Tribe, Jenny Carter, Sergio A. Silverio, The UK Preterm Clinical Network

**Affiliations:** ^1^ Department of Psychology, Institute of Population Health, University of Liverpool, Liverpool, United Kingdom; ^2^ Department of Women & Children’s Health, School of Life Course & Population Sciences, King’s College London, London, United Kingdom; ^3^ Fetal Medicine Unit, Liverpool Women’s NHS Foundation Trust, Liverpool, United Kingdom; ^4^ Fetal Medicine Unit, St. George’s University Hospitals NHS Foundation Trust, London, United Kingdom

**Keywords:** antenatal anxiety, antenatal depression, preterm birth, pregnancy, wellbeing, mental health

## Abstract

**Introduction:**

Women identified at risk for preterm may be vulnerable to developing mental health difficulties due to the increased likelihood of poor pregnancy outcome and uncertainty surrounding their delivery. Formal assessment of mental wellbeing in specialist preterm birth clinics is not routinely offered, but may offer the opportunity for early intervention.

**Methods:**

We aimed to investigate if demographic characteristics and obstetric risk factors were associated with psychological wellbeing in women at risk of preterm birth. We explored associations between mental wellbeing and risk factors for preterm birth using hierarchical regression analyses.

**Results:**

When demographic variables were considered alone, high body mass index (BMI) was significantly associated with anxiety (*p* = .026), however became non-significant when obstetric risk factors were also considered. Previous late miscarriage was associated with high anxiety (*p* = .049). Lower maternal age at estimated date of delivery (*p* = .019) and non-European ethnic heritage (*p* = .029) were significantly associated with depression. High maternal BMI (*p* < .001), being of any other non-European ethnic heritage (*p* = .043), currently smoking (*p* = .002), and previous spontaneous preterm birth (*p* = .017) were associated with lower perceived wellbeing.

**Discussion:**

The results of this study highlight the importance of routinely monitoring mental health in women with relevant risk factors, particularly if they are already at risk of preterm birth.

## 1 Introduction

Preterm birth (PTB; <37 weeks' gestation) is the leading cause of death amongst children under five, and survivors can experience significant short- and long-term morbidities (1, 2). It is suggested that one-in-four women have clinically relevant symptoms of anxiety during pregnancy, which may increase the likelihood of preterm labour and birth (3). Antenatal symptoms of depression have also been linked with increased likelihood of PTB (4). Other factors associated with increased risk of preterm birth include demographic and lifestyle factors (e.g., ethnicity (5); exposure to smoking (6); advanced maternal age (7); low (8) and high (9) body mass index [BMI], domestic abuse during pregnancy (10)) as well as medical and obstetric history [e.g., invasive cervical surgery, previous PTB; previous miscarriage (11)].

Recent research focusing on fetal programming highlights the need to consider maternal well-being during pregnancy as this can affect the baby before and after birth (12). Women identified at risk for PTB may be particularly vulnerable to developing anxiety and depression due to the increased likelihood of poor pregnancy outcome, uncertainty surrounding their delivery, and the infant's safety (13). Research investigating psychological wellbeing specifically in women at risk of PTB is limited. Most studies are predominantly qualitative in nature, but do link maternal anxiety with PTB and the associated treatments. Research suggests women are positive about interventions to try and reduce its likelihood (14); and longitudinal work suggesting women receiving care for PTB demonstrated high levels of anxiety and depression which improved over time, as did satisfaction with care (15).

Women already at higher risk of preterm birth may be at even greater risk if they suffer from poor mental health, so screening and treating this could improve outcomes. A recent review highlights the need to explore women's wellbeing and mental health when under the care of such specialist antenatal clinics (16). Specialist PTB clinics provide additional pregnancy care for women at risk which includes monitoring, e.g., cervical length measurement, and interventions e.g., cerclage and progesterone supplementation, when necessary. Although clinicians are sensitive to the psychological needs of their patients, formal assessment of mental wellbeing is not routinely offered. We used data from the implementation of mental wellbeing screening at one London hospital to investigate if demographic characteristics and obstetric risk factors were associated with psychological wellbeing in women at risk of PTB.

## 2 Participants, ethics and methods

### 2.1 Ethical approval

Data for this study were obtained from the UK Preterm Clinical Network (PCN) database [NHS Research Ethics Committee (REC) Reference:16/ES/0093] (17) following submission of an application that was reviewed and approved by the PCN Database Access Committee in February 2023.

### 2.2 Participants and recruitment

In one London hospital, mental wellbeing screening was introduced as part of King's Health Partners' Integrating Mental & Physical Healthcare: Research, Training & Services (IMPARTS) programme (18).

On arrival at the preterm clinic, women were asked to complete an electronic mental wellbeing screening questionnaire prior to their consultation. The questionnaire included questions from the GAD-7 (19), PHQ9 (20) and PROMIS-10 (21) validated instruments. Results (and associated scores) are transferred directly to the hospital electronic patient record, where they are reviewed by the attending clinician. Additional support or referral to specialist services is offered as required. The screening scores are also entered onto the Preterm Clinical Network (PCN) Database, subject to patient consent, which is a repository for specialist preterm clinical data.

The data can be used for local audit and wider cohort studies that are approved by the PCN Database Access Committee. Data were extracted for participants who were eligible for inclusion in the study through attendance to the London NHS Trust who were collecting mental wellbeing data from their PTB clinic patients.

All patients attending the clinic and consenting to their data being held on the PCN Database between 1st July 2021 and 5th April 2023 were included. This is the date range that the IMPARTS wellbeing screening was introduced. All women who consented to their data being included in the PCN database are presented for descriptive purposes, but only participants who provided scores on either measure of psychological wellbeing (anxiety, depression, and perceived wellbeing), as well as the demographic and obstetric characteristics outlined in the method of analysis were included in further analysis. Complete data were available for *n* = 251 (GAD-7), *n* = 245 (PHQ-9), and *n* = 245 (PROMIS-10).

### 2.3 Measures

#### 2.3.1 Demographic and obstetric characteristics

Demographic characteristics were obtained from participants, including estimated age at delivery, heritage, and smoking status. Obstetric characteristics included gravida, parity, previous late miscarriage, and previous spontaneous PTB. See Table 1 for full details.

**Table 1:** Maternal demographic and obstetric characteristics.

Variable^a^	Value
Gravida (M ± SD) *N* = 662	3.08 ± 1.93
Parity (*N*/%) *N* = 662	
Primiparous	274 (41.45%)
Multiparous	388 (58.55%)
BMI (M ± SD) *N* = 647	25.66 ± 5.72
Heritage (*N*/%) *N* = 648	
African	78 (12.04%)
AfroCaribbean	43 (6.64%)
Bangladeshi	11 (1.70%)
European	404 (62.35%)
Far East Asian	21 (3.24%)
Indian	11 (1.70%)
Middle Eastern	5 (0.77%)
Pakistani	4 (0.62%)
South American	12 (1.85%)
South East Asian	3 (0.46%)
Unclassified (other)	44 (6.79%)
Unknown	12 (1.85%)
Smoking Status (*N*/%) *N* = 646	
Current	24 (3.72%)
Ex-Smoker (gave up before pregnancy)	73 (11.30%)
Ex-Smoker (gave up during pregnancy)	10 (1.55%)
Never smoked	538 (83.28%)
Unknown	1 (0.15%)
Maternal age at EDD (Median/Range) *N* = 658^b^	35.00 (51.00)
Country of Birth (*N*/%) *N* = 629	
United Kingdom	352 (55.96%)
United States	17 (2.70%)
Italy	20 (3.18%)
Spain	12 (1.91%)
Nigeria	12 (1.91%)
Jamaica	12 (1.91%)
Ghana	12 (1.91%)
France	11 (1.75%)
Other European Country	71 (11.29%)
Other Non-European Country	110 (17.49%)
Primary Language (*N*/%) *N* = 633	
English	517 (81.67%)
Spanish	20 (3.16%)
Italian	19 (3.00%)
Portuguese	13 (2.05%)
Other European Language	46 (7.27%)
Other Non-European Language	18 (2.84%)
Previous Spontaneous Preterm Birth (*N*/%) *N* = 663	
Yes	123 (18.55%)
No	540 (81.45%)
Number of Previous Spontaneous Preterm Birth (*N*/%)^b^ *N* = 122	
One	105 (86.07%)
Two	15 (12.30%)
Three	2 (1.64%)
Most Significant Gestation of Previous Spontaneous Preterm Birth (M ± SD)^c^ *N* = 122	28.23 ± 4.87
Previous Cervical Surgery (*N*/%) *N* = 663	
Yes	281 (42.38%)
No	382 (57.62%)
Number of Previous Cervical Surgeries (*N*/%)^b^ *N* = 280	
One	237 (84.64%)
Two	39 (13.93%)
Three or more	4 (1.43%)
Most Significant Cervical Procedure (*N*/%)^b^ *N* = 278	
LLETZ	232 (83.45%)
Cone	41 (14.75%)
Trachelectomy	2 (0.72%)
Unknown	3 (1.08%)
Uterine Abnormality (*N*/%) *N* = 663	
Yes	51 (7.69%)
No	612 (92.31%)
Details of Uterine Abnormality (*N*/%)^b^ *N* = 50	
Arcuate Uterus	4 (8.00%)
Bicornuate Uterus	21 (42.00%)
Didelphic	5 (10.00%)
Fibroids	1 (2.00%)
Resection of Uterine Septum	3 (6.00%)
Septum (Septate)	9 (18.00%)
Unicornuate Uterus	7 (14.00%)
Previous PPROM (*N*/%) *N* = 663	
Yes	87 (13.12%)
No	576 (86.88%)
Number of Previous PPROM (*N*/%)^c^ *N* = 86	
One	75 (87.21%)
Two	6 (6.98%)
Three	5 (5.81%)
Most Significant Gestation of Previous PPROM (M ± SD)^c^ *N* = 83	25.65 ± 5.07
Previous Late Miscarriage (*N*/%) *N* = 663	
Yes	133 (20.06%)
No	530 (79.94%)
Number of Previous Late Miscarriages (*N*/%)^c^ *N* = 133	
One	107 (80.45%)
Two	19 (14.29%)
Three or more	7 (5.26%)
Most Significant Gestation of Previous Late Miscarriage (M ± SD)^c^ *N* = 130	19.79 ± 3.20
Multiple Pregnancy (*N*/%) *N* = 663	
Yes	34 (5.13%)
No	629 (94.87%)
Gestational Age (in weeks) at first visit (Median/Range)^d^ *N* = 652	18.00 (60.00)
PHQ-9 Total (M ± SD) *N* = 283	2.07 ± 4.75
GAD-7 Total (M ± SD) *N* = 289	2.96 ± 5.21
PROMIS-10 Total (M ± SD) *N* = 282	34.63 ± 6.17

^a^ Please note that due to missing data, the *N* value for each variable differs. The *N* value is therefore listed before each variable. Due to rounding, not all percentages add to 100%.

^b^ One participant had their age listed as 0 and two had their age listed as 213 and 214. The former was removed both from this analysis and the inferential analysis, whereas the latter had their age coded as missing but were not included in any subsequent analyses.

^c^ Only considers those who indicated yes to the previous question.

^d^ Five participants had impossible values for this variable, so were not included here.

#### 2.3.2 Generalized anxiety disorder 7-item (GAD-7)

The GAD-7 is a brief, 7-item measure assessing symptoms of generalised anxiety disorder during the previous 14-days, on constructs such as excessive worry and fear of something awful happening (19). Participants score on a Likert scale from 0 (not at all) to 3 (nearly every day). The measure has been validated for use in pregnant women and demonstrates good diagnostic accuracy (22). Higher scores indicate higher anxiety.

#### 2.3.3 Patient health questionnaire 9-item (PHQ-9)

The PHQ-9 assessing symptoms of depression during the previous seven days on a Likert scale from 0 (not at all) to 3 (nearly every day) (20). The measure has been validated in pregnant women (23). Higher scores indicate higher symptoms of depression.

#### 2.3.4 Patient reported outcomes measurement information system 10-item (PROMIS-10)

The PROMIS-10 is a 10-item self-report measure of perceived quality of life, mental and physical health (21). Participants rate questions from 1 (poor) to 5 (excellent). The measure has been widely used in pregnant women (24). Lower scores indicate lower perceived wellbeing.

### 2.4 Method of analysis

Data were analysed using three hierarchical multiple linear regressions for each outcome variable (GAD7, PHQ9, and PROMIS-10 scores). Analysis was conducted on complete cases for the psychological variable of interest and demographic and obstetric characteristics being controlled for. All the initial linear models had substantial issues with heteroskedasticity so a Box-Cox transformation (25), ascertained as the appropriate method using the gamlss (17) package in R, was conducted on the dependent variables (with subsequent models demonstrating homoskedasticity). The following demographic variables were added as step one in the regression models, decided *a priori* as previous literature has indicated they may increase the likelihood of PTB as outlined above; age at estimated date of delivery [EDD] (continuous), BMI (continuous), heritage (0 = European heritage, 1 = any other non-European ethnic background) and smoking status (0 = currently smoking, 1 = never/ex-smoker). For purposes of analysis, ethnicity was treated as binary. As most participants were of European heritage, it was deemed most appropriate to split in the above manner. The following obstetric characteristics were added as step two to the models; parity (continuous), previous late miscarriage (0 = yes, 1 = no) and previous spontaneous PTB (0 = yes, 1 = no). Only participants who had complete data for all of the demographic and obstetric risk factors for each mental health outcome were included in the analysis.

## 3 Results

### 3.1 Participants

Participants (*N* = 663; *Median_Age_* = 35.00 years) were predominantly of European heritage (62.35%) and born in the United Kingdom (55.96%), with only a minority being ex-smokers who gave up before or during pregnancy (12.85%) and most having never smoked (83.28%). A small number of women had experienced previous spontaneous PTB (18.55%), nearly half (42.38%) had previously had cervical surgery, and very few women had previous experience of preterm premature rupture of the membranes (PPROM; 13.12%). See Table 1 for full characteristics.

### 3.2 Anxiety

Step one (age at EDD, BMI, heritage, smoking status), was significant and predicted approximately 4% of variance in anxiety scores [*R*^2^ = 0.04, F(4,245) = 2.76, *p* = .028]. Only maternal BMI was a significant individual predictor (B = 0.01, SE = 0.01, 95% CI = 0.002 to 0.024, *p* = .026), with higher BMI being associated with higher levels of anxiety.

Step two (parity, previous late miscarriage, previous spontaneous preterm birth) predicted an additional 2% of variance but was not significant [*R*^2^-change = 0.02, F-change (3, 242) = 1.68, *p* = .173]. Only previous late miscarriage was a significant individual predictor (B = −0.17, SE = 0.08, 95% CI = −0.333 to −0.001, *p* = .049), with experience of a previous late miscarriage being associated with higher anxiety (see Table 2).

**Table 2:** Hierarchical linear regression models predicting GAD-7 scores. Values represent unstandardised coefficients.

	Cumulative	F-change (df)	Simultaneous	95% CI	*p*-value
	*R*^2^-change		*Β* (SE)		
Step 1	0.04	2.76 (4, 245)*			
Age at Estimated Date of Delivery			−0.01 (0.01)	−0.023 to 0.005	.204
BMI			0.01 (0.01)	−0.001 to 0.022	.066
Ethnic Heritage			−0.03 (0.07)	−0.173 to 0.123	.739
Smoking Status			−0.07 (0.21)	−0.495 to 0.345	.725
Step 2	0.02	1.68 (3, 242)			
Parity			0.00 (0.04)	−0.076 to 0.083	.935
Previous Late Miscarriage			−0.17 (0.08)	−0.333 to −0.001	.049
Previous Spontaneous Preterm Birth			−0.10 (0.09)	−0.281 to 0.077	.265

* *p* < .05.

** *p* < .01.

*** *p* < .001.

### 3.3 Depression

Step one (age at EDD, BMI, heritage, smoking status) significantly predicted approximately 9% of variance in depression scores [*R*^2^ = 0.09, F(4,239) = 5.77, *p* < .001]. Age at estimated date of delivery (EDD; B = −0.01, SE < 0.01, 95% CI = −0.019 to −0.002, *p* = .011) and heritage (B = 0.12, SE = 0.04, 95% CI = 0.032 to 0.200, *p* = .007) were significant individual predictors, with those of any other non-European heritage and younger age having higher PHQ-9 scores.

Step two (parity, previous late miscarriage, previous spontaneous preterm birth) predicted an additional 1% of variance *R*^2^-change = 0.01, F-change (3, 236) = 0.54, *p* = .658, but this was non-significant. As can be seen in Table 3, age at EDD (B = −0.01, SE < 0.01, 95% CI = −0.019 to −0.002, *p* = .019) and heritage (B = 0.10, SE = 0.05, 95% CI: 0.011 to 0.191, *p* = .029) remained significant individual predictors with those of any other non-European heritage and younger age having higher PHQ-9 scores.

**Table 3:** Hierarchical linear regression models predicting PHQ-9 scores. Values represent unstandardised coefficients.

	Cumulative	F-change (df)	Simultaneous	95% CI	*p*-value
	*R*^2^-change		*Β* (SE)		
Step 1	0.09	5.77 (4, 239)***			
Age at Estimated Date of Delivery			−0.01 (<0.01)	−0.019 to −0.002	.019
BMI			<0.01 (<0.01)	−0.003 to 0.011	.226
Ethnic Heritage			0.10 (0.05)	0.011 to 0.191	.029
Smoking Status			−0.17 (0.13)	−0.422 to 0.089	.202
Step 2	0.01	0.54 (3,236)			
Parity			0.01 (0.02)	−0.043 to 0.055	.806
Previous Late Miscarriage			−0.06 (0.05)	−0.160 to 0.042	.253
Previous Spontaneous Preterm Birth			0.02 (0.06)	−0.089 to 0.133	.698

* *p* < .05.

** *p* < .01.

*** *p* < .001.

### 3.4 Perceived wellbeing

Step one (age at EDD, heritage, smoking status) significantly predicted approximately 21% of variance in PROMIS-10 scores [*R*^2^ = 0.21, F(4,239) = 16.11, *p* < .001]. Maternal BMI (B = −11.32, SE = 2.14, 95% CI = −15.530 to −7.110, *p* < .001), heritage (B = −74.52, SE = 26.13, 95% CI = −126.000 to −23.037, *p* = .005), and smoking status (B = 237.61, SE = 78.95, 95% CI = 82.097 to 393.129, *p* = .003) were significant individual predictors, with those who were ex- or never-smokers having higher PROMIS-10 scores.

Step two (parity, previous late miscarriage, previous spontaneous preterm birth) significantly predicted an additional 3% of variance *R*^2^-change = 0.03, F-change (3, 236) = 3.55, *p* = .015. As can be seen in Table 4, high BMI (B = −10.60, SE = 2.13, 95% CI = −14.799 to −6.401, *p* < .001, any other non-European ethnic heritage (B = −56.55, SE = 27.78, 95% CI = −111.270 to −1.822, *p* = .043) and currently smoking status (B = 240.34, SE = 78.27, 95% CI = 86.139 to 394.545, *p* = .002) remained significant individual predictors and were associated with lower perceived wellbeing. Previous spontaneous PTB was also a significant predictor (B = 80.87, SE = 33.52, 95% CI = 14.845 to 146.901, *p* = .017), with prior spontaneous preterm birth being associated with lower wellbeing.

**Table 4:** Hierarchical linear regression models predicting PROMIS-10 scores. Values represent unstandardised coefficients.

	Cumulative	F-change (df)	Simultaneous	95% CI	*p*-value
	*R*^2^-change		Β (SE)		
Step 1	0.21	16.11 (4, 239)***			
Age at Estimated Date of Delivery			1.68 (2.62)	−3.484 to 6.839	.523
BMI			−10.60 (2.13)	−14.799 to −6.401	<.001
Ethnic Heritage			−56.55 (27.78)	−111.270 to −1.822	.043
Smoking Status			240.34 (78.27)	86.139 to 394.545	.002
Step 2	0.03	3.55 (3, 236)*			
Parity			−14.81 (15.07)	−44.508 to 14.886	.327
Previous Late Miscarriage			12.34 (31.52)	−49.768 to 74.438	.696
Previous Spontaneous Preterm Birth			80.87 (33.52)	14.845 to 146.901	.017

* *p* < .05.

** *p* < .01.

*** *p* < .001.

## 4 Discussion

### 4.1 Summary of main findings

This study aimed to investigate if demographic and obstetric characteristics were associated with increased likelihood of mental health difficulties in high risk pregnant women attending a specialist PTB surveillance clinic. In terms of anxiety, when demographic variables were considered alone, higher maternal BMI was a significant individual predictor. However, the overall model became non-significant when obstetric characteristics were also considered. Only previous late miscarriage was significantly, negatively associated with symptoms of anxiety. Maternal age at EDD and ethnic heritage were significantly associated with depression at both steps; with younger women of any other non-European heritage having higher levels of depressive symptomatology. Finally, when considering perceived wellbeing: high BMI, any other non-European ethnic heritage, currently smoking, and previous experience of a spontaneous PTB were associated with lower PROMIS-10 scores.

### 4.2 Strengths and limitations

This study utilises clinical data prospectively collected for the PCN database from a PTB surveillance clinic in London, United Kingdom, allowing for unique insight into ethnically diverse data derived from a region of high levels of social complexity and a hospital clinic which serves women with varying degrees of social deprivation. The inclusion of women from a wide range of ethnicities is a particular strength, especially when these groups remain largely understudied and therefore under-represented in research (26). However, the study is limited by the use of generalised measures of anxiety and depression, which do not capture emotions unique to pregnancy. It may be that results may be different if a pregnancy-specific measure was used, particularly as previous studies (27) have suggested that weight and smoking status are associated with pregnancy-specific anxiety when they were not in this study. Future studies should endeavour to use pregnancy-specific measures, particularly as pregnancy-specific anxiety measured using these instruments (28) is distinct from generalised anxiety, and women who do not meet clinical anxiety criteria will go under-recognised despite having pregnancy-specific anxiety. Furthermore, the ability to offer specifically targeted interventions as measured in these scales may be missed.

### 4.3 Interpretation of study findings and comparison with published evidence

In the United Kingdom, approximately 50% of pregnant women are overweight or living with obesity (29), and it is well-established that these women are at increased risk for poor mental health outcomes like anxiety when compared to pregnant women of a healthy weight (30). Women report feelings of stigma surrounding their pregnancy and highlight anxiety surrounding the medicalisation of a “high-risk” pregnancy (30). Women who have been identified as at high risk of PTB are already more likely to experience anxiety, but this is further compounded if they are also overweight or living with obesity (9). However, Tsur et al. (31) describes an “*obesity paradox*” whereby women living with obesity with no comorbidities had a lower associated risk ratio of spontaneous PTB compared to healthy controls, suggesting that other factors must also be considered alongside BMI. This may also go some way to explaining the non-significant finding in the current study when both obstetric and demographic variables were considered together. Previous late miscarriage, however, was significantly associated with anxiety. This is consistent with a wealth of literature that demonstrates previous miscarriage is related to anxiety and fear of childbirth during pregnancy (32, 33).

It has been suggested that hormonal changes associated with depression may lead to PPROM, which can, in turn, result in PTB (34). Previous studies show an association between younger age and symptoms of antenatal depression (35, 36), perhaps due to the likelihood of reduced social support and financial instability in younger women. Given that younger women under 18 years of age are also at increased risk of preterm delivery (37), this group may require focused attention if they also experience are at increased risk of depressive symptoms. Qualitative studies investigating why this may be the case are scarce, however a recent study of women during the perinatal period highlights feelings of loneliness, shame, and isolation in younger participants, particularly as they feel they are going to be labelled as a “bad” mother, leading to feelings of depression (38). Racial and ethnic differences when considering risk of PTB are well-known, with studies indicating that African American (39) and Asian (40) populations with depressive symptoms are at increased risk of preterm birth. However, some studies have found no increased risk of PTB in other minority ethnic groups (40). Women from ethnic minority backgrounds can face several barriers to seeking mental health treatment, including stigma and low levels of social support (41). Research shows that there is a link between ethnicity and BMI; South Asian women have a higher risk of complications during pregnancy relating to obesity compared to white British women (42). As they are more likely to experience poor pregnancy outcomes, including preterm birth, it is important to ensure all women from vulnerable groups, including those from non-white ethnic minority and socially deprived groups, receive the same standard of care offered to all pregnant women. This involves a concerted effort to reach women from underserved communities, e.g., following up those not attending antenatal appointments and providing interpreting services for non-English speakers.

Smoking during pregnancy not only presents risks to the developing infant, including low birth weight and restricted growth in-utero (43), but also to the mother, including depressive symptoms being associated with nicotine addiction (44), especially in mothers of low socio-economic status (45). Studies have demonstrated that good mental health during pregnancy can be associated with smoking cessation (46). Women who smoke during pregnancy have reported feelings of stigmatisation and “othering”, so are reluctant to disclose this information or seek help to stop (47). This is mirrored by the findings of Stacey et al. (48) where women highlight the need for more information about the risk of smoking during pregnancy and the need for a non-judgmental approach.

Previous spontaneous mid-trimester loss (miscarriage between 14 and 23 weeks') and PTB are significant risk factors for subsequent preterm delivery (8, 49–51). Women who experience an increase in anxiety, but not depression, during their pregnancy are at increased likelihood of preterm birth (52), and this is important to consider in the context of the current study, where it is well established that being labelled “at-risk” can increase anxiety. This is especially important to consider in women who have been referred to a PTB clinic as, although previous spontaneous PTB can increase anxiety in a subsequent pregnancy, many women can see a reduction in their anxiety symptoms as they perceive their care as good (15). However, it is important to consider that although the results of the current study can, in part, be related to feelings relating to being labelled “at-risk”, many women under the care of specialist PTB clinics embrace this label and see it as a positive (14). The reassurance that women can receive by attending a specialist preterm clinic may go some way to reducing their anxiety, which could be considered a preterm prevention intervention, alongside others, such as cerclage and progesterone supplementation. As outlined by O'Brien et al. (14), Williams and Mackey's (53) “*Women's experiences of preterm labor: a feminist critique*” argues that being labelled “at-risk” means women at risk of preterm labour can assign the blame they would usually inflict upon themselves to healthcare professionals, and so are more likely to follow medical advice. This is especially important to consider in women who have previous experience of PTB, as feelings of guilt and blame may lead to hypervigilance and overprotection in subsequent pregnancies (54), which can be anxiety inducing.

### 4.4 Conclusions

To conclude, the results of the current study demonstrate that a high BMI is associated with increased anxiety when demographic factors are considered alone, as is previous late miscarriage; younger maternal age and non-European ethnic heritage were significantly associated with depression, and high BMI, non-European ethnic heritage, currently smoking, and previous experience of a spontaneous PTB were associated with lower perceived wellbeing. These results highlight the need for healthcare professionals to monitor mental health in all pregnant women, but particularly those with these risk factors, in a sensitive and non-judgmental manner, and to do what they can to offer appropriate support and referral to mental health services when required. A mental wellbeing screening programme, such as described in this paper, embedded with specialist preterm services, may help clinicians to identify those most in need of additional support.

## Data Availability

The datasets presented in this article are not readily available because Data are available upon reasonable request from The Preterm Clinical Network (PCN) Database, and subject to review by the PCN Database Access Committee. Requests to access the datasets should be directed to Jenny.Carter@kcl.ac.uk.
